# Seeds attached to refrigerated shipping containers represent a substantial risk of nonnative plant species introduction and establishment

**DOI:** 10.1038/s41598-020-71954-3

**Published:** 2020-09-14

**Authors:** Rima D. Lucardi, Emily S. Bellis, Chelsea E. Cunard, Jarron K. Gravesande, Steven C. Hughes, Lauren E. Whitehurst, Samantha J. Worthy, Kevin S. Burgess, Travis D. Marsico

**Affiliations:** 1 United States Department of Agriculture, Forest Service, Southern Research Station, Athens, GA USA; 2 Arkansas Bioscience Institute and Department of Computer Science, Arkansas State University, Jonesboro, AR USA; 3 Department of Biological Sciences, Arkansas State University, Jonesboro, AR USA; 4 Department of Plant Biology, The Herbarium at the University of Georgia, Athens, GA USA; 5 Department of Biology, Columbus State University, Columbus, USA; 6 Present Address: Department of Plant Pathology, University of Georgia, Athens, GA USA; 7 Present Address: Department of Biology, University of Florida, Gainesville, FL USA; 8 Present Address: Department of Biology, University of Maryland, College Park, Maryland USA

**Keywords:** Ecological modelling, Invasive species, Ecology, Plant sciences, Plant ecology, Environmental impact

## Abstract

The initial processes for successful biological invasions are transport, introduction, and establishment. These can be directly influenced or completely avoided through activities that reduce the number and frequency of entering nonnative propagules. Economic and environmental benefits through preventative monitoring programs at early stages of invasion far outweigh the long-term costs associated with mitigating ecological and economic impacts once nonnative species establish and spread. In this study, we identified 30 taxa of hitchhiking plant propagules on the air-intake grilles of refrigerated shipping containers arriving into a United States seaport from a port on the Pacific coast of South America. The four monocotyledonous taxa with the highest number of seeds collected were analyzed; we estimated propagule pressure, germination, and survivorship of these taxa, and we used the estimates to determine likelihood of establishment. At the levels of propagule pressure estimated here, non-zero germination and survival rates resulted in high establishment probabilities even when escape rates from shipping containers were modelled to be exceedingly low. Our results suggest high invasion risk for nonnative taxa including *Saccharum spontaneum* L., a listed Federal Noxious Weed. Currently, not all shipping containers arriving at USA ports are thoroughly inspected due to limited personnel and funding for biological invasion prevention. Our results indicate that there is a significant risk from only a few propagules escaping into the environment from this source, and we propose possible solutions for reducing this risk.

## Introduction

Biological invasions continue to accelerate in frequency and number with the increasing volume of economic trade^1^. The negative economic and ecological costs resulting from biological invasions are often substantial and difficult to manage or even contain once impacts occur^2^. Moreover, funding for the prevention, containment, or general management of invasive organisms and pathogens is not adequate and continues to be substantially reduced since the mid-2000s^3,4^. One of the major issues with nonnative invasive species is that once negative impacts are recognized, the organisms have already passed through the filters of transport, introduction, successful establishment, and spread in the novel geographic range^5^. Investment in the prevention and early detection of nonnative taxa with known negative impacts results in nearly a 100-fold increase of economic return when compared to managing widespread taxa that can no longer be contained (see: Generalised Invasion Curve^2^). Therefore, strategic prevention of entry and reductions in establishment probabilities of nonnative propagules, especially those that are characterized as high-risk, would be economically and environmentally beneficial in an era of high-volume and high-frequency trade.

Propagules are individuals or parts of biological organisms that may give rise to another individual (e.g., eggs, larvae, seeds, fragments); these propagules are generally smaller than the parent/source material and are more likely to be inadvertently transported in larger volumes (i.e., propagule size)^6,7^. During the early stage of the invasion process, propagule pressure is considered one of the most important contributing factors to successful biological invasions of diverse taxa^6–10^. Propagule pressure is empirically derived from the number of viable propagules (i.e., propagule size equal to the number of arriving propagules^6,8–10^) multiplied by the frequency of introductions^6,11^. In a broader context, propagules are biological structures that give rise to separate individuals and are migrants into a population over a defined period of space and time^12^. Propagule pressure is considered the only factor that can influence both biotic and abiotic factors of the naïve environment and may modulate and influence invasion success or failure^8^. Therefore, for any models developed to assess establishment risk of invasive species, the propagule pressure must be parameterized effectively^9,13^.

One mechanism for arrival of propagules of nonnative species is through oceanic commercial shipping that now occurs globally. Numbers and volumes of shipping containers continue to increase in the anthropogenic commodity trade, and these include everything from new shoes to frozen seafood to fresh fruits^14^. Almost all ordinary consumer commodities are sea-transported on vessels that move hundreds to thousands of shipping container volumes per year at any single port site. Refrigerated shipping containers are built with climate-control capacities to hold the sealed container at specific temperatures for the desired commodity being transported, and all climate-controlled shipping containers possess condensers and air-intake grilles. Air-intake grilles are key areas where hitchhiking propagules may attach and be transported across continents to potentially hospitable new environments.

Here, we focus on inadvertent, passive hitchhiking plant propagules associated with global trade, for which over 90% is conducted by international sea transportation^1,15^. As a result, seaports intuitively serve as the initial terrestrial sites of entry for nonnative species. It should be noted, however, that the actual journey of propagules in the global sea transportation network is variable and is subject to pressures that may reduce propagule quality and viability^12,16^. Therefore, the risk associated with these propagules remains unknown. For many passively dispersing propagules, such as plant fruits or seeds, the probability of surviving transport on the vector (i.e., the shipping container) and the subsequent ability to disembark and establish onto terrestrial substrate could already be diminished. Propagule pressure and correspondingly, invasion risk, remains difficult to quantify^17^. Of significant interest to federal regulatory and enforcement agencies is the viability of plant propagules (including dispersed fruits and seeds, hereafter called “seeds” for simplicity^9^) hitchhiking specifically on refrigerated shipping containers that generally transport commodities that fall within the jurisdiction of the Agriculture Inspection Program(s) executed by United States Customs & Border Protection (USCBP, US Dept. of Homeland Security). The particular concern expressed to researchers was the possibility of encountering any of the 112 Federal Noxious Weeds (FNWs), known “high-risk” plant species, or those that are already invasive in the USA that pose significant and immediate threats to the agro-security of the nation and are viable propagules^18^. For those FNWs that are already established in the USA, additional propagule pressure may contribute to increased genetic diversity, distribution, abundance, and other impacts, thereby providing an opportunity for increased invasiveness over time^5^.

We developed this study to focus on air-intake grilles of refrigerated shipping containers containing an agricultural commodity at the container terminal at the Port of Savannah, Georgia, USA (Supplementary Fig. 1). The shipping route is generally known to originate on the western seaboard of South America (eastern Pacific), pass through the Panama Canal, where the refrigerated shipping containers may remain at a port for 16–72 h before continuing the journey that deposits the cargo at the Port of Savannah (western Atlantic). This process includes two different cargo ships for inter-oceanic commodity transport and is referred to as “trans-shipping.” Therefore, seeds may be picked up at the farm-of-origin, port-of-origin, during trans-shipment at the canal, or anywhere along the way; though, it should be noted that the highest probability of obtaining nonnative, high-risk, ruderal, abundant plant seeds would be on land^19^. We present our assessment of the plant propagule abundance, frequency, and diversity collected from refrigerated shipping containers arriving at the Port of Savannah during two trade seasons (August-February 2015–2016 [Season 1] and August-February 2016–2017 [Season 2]).

**Figure 1:**
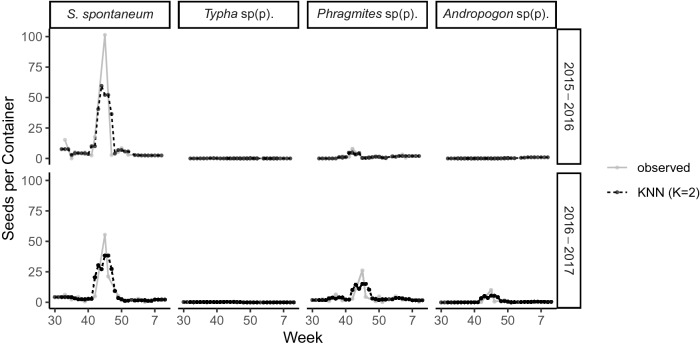
Observed and estimated seeds per container (i.e., propagule size) for four monocotyledonous focal taxa over time (shown as week of the year) in Season 1 and Season 2. Estimations of seeds per containers were based on *K*-Nearest Neighbors regression with *K* = 2. Created in R version 3.5.1^44^.

From Season 2, we also provide details on seed germination and survivorship of seedlings of four focal monocotyledonous taxa to evaluate propagule pressure and risk of establishment. Using our empirically derived estimates of propagule pressure, germination, and survivorship, we then developed process-based simulation models to estimate the risk of nonnative species at what we consider the first terrestrial substrates available to these passive hitchhikers into the USA (around the final port of entry). Process-based simulation models have been shown to be one of the most useful frameworks in botanical epidemiology for cohesive synthesis of empirical estimates from different experiments^20^. These models are one of the few ways to ask “if–then” questions about how changes in driving variables could impact future behaviour of complex systems^21^. With many parallels between the processes of epidemiology and biological invasion^22^, extension of botanical epidemiological models is a promising avenue to explore research questions in invasion ecology, especially propagule pressure and preventability. We developed simple process-based simulation models to evaluate the prediction that seeds hitchhiking on shipping containers pose a substantial invasion risk at international points-of-entry. Our major research objective was to empirically assess nonnative plant propagule pressure (via seeds) in real-time, based on a strategic sampling interval and approach (see Methods). This influx of nonnative propagules into a single receptor locality, the Garden City Terminal (GCT, hereafter), which is the container handling receptor facility at the Port of Savannah, allowed us to assess propagule pressure over two seasons of collected data and then evaluate the risk of establishment based on variable rates of escape to create our risk models. We aim to assess risk of nonnative propagule pressure into an international trade hub, at which if the nascent invasive species becomes primarily established, it may also then secondarily spread into other areas, including agricultural areas and areas of biodiversity and conservation concern.

## Results

### Propagule numbers and diversity

In Season 1, we sampled 5,537 seeds originally sorted into 27 morphotypes. In Season 2, 5,509 seeds were sampled and originally sorted into 44 morphotypes, with some of the morphotypes being shared between the two sampling seasons (Table S3; 59 total morphotypes over the two sampling seasons). Subsequent morphological identification of pre-sorted morphotypes resulted in many of the morphotypes being combined, such that across both sampling seasons, a total of 22 unique plant taxa (identified to the family, tribe, genus, or species level) were identified for inclusion in germination trials (Taxa 1–22, Table S4). These taxa represented at least 30 unique species, though a few were difficult to distinguish from seed characteristics and were combined into the 22 taxa for germination trials (Table S4). All taxa identified were from five vascular plant families (Asteraceae, Brassicaceae, Cyperaceae, Poaceae, and Typhaceae; Table S3, S4), most of which are known to have lightweight, wind-dispersed seeds.

We selected the four monocotyledonous taxa with the largest number of sampled seeds as focal taxa for the remainder of the study: *Saccharum spontaneum* L., *Typha* sp(p)., *Phragmites* sp(p)., and *Andropogon* sp(p). (Note that since it was uncertain if the seeds of *Typha*, *Phragmites*, and *Andropogon* represented a single or multiple species, we consistently use the abbreviation sp(p). henceforth.) The influx of seeds for all four focal taxa was heterogeneous over time, peaking in mid-November for three of the taxa (Fig. 1). There were differences between the two sampling seasons, with *Andropogon* sp(p). being much more abundant in Season 2 than Season 1 (Fig. 1). *Phragmites* sp(p). showed a shift in the peak arrivals a few weeks later in Season 2 when compared to Season 1 (Fig. 1). Also, the seasonality of arrivals of seeds per container were independent of the number of containers arriving and the sampling approach (compare arrivals and sampling effort from Supplementary Fig. 2 with seeds per container in Fig. 1 on the same time scale).

### Germination and survivorship trials

From the 22 taxa sorted for use in germination trials, 12 taxa had samples of seeds collected in Season 2 to initiate trials (Table S4). Of these, 9 taxa (75%) successfully germinated, with rates ranging from 9–40% per species (mean = 25%, SD = 11.2% excluding the three taxa with unsuccessful germination: two taxa in the Asteraceae family and one in the Poaceae family). Of our four focal taxa, the one FNW, *Saccharum spontaneum*, had 9% germination (160 seedlings from 1,690 seeds), *Typha* sp(p). exhibited 18% germination (164 seedlings from 923 seeds), *Phragmites* sp(p). had 26% germination (180 seedlings from 697 seeds), and *Andropogon* sp(p). exhibited 37% germination (63 seedlings from 170 seeds) (Table 1).

**Table 1:** Values used for all parameters in simulation models. Germination and survival are based on empirical estimates, though we also modeled lower survival rates.

Species	Estimated Total Seeds		Observed Total Seeds		Germination (%)	Survival (%)	Reproduction (Seeds)
	Season 1	Season 2	Season 1	Season 2			
*Saccharum spontaneum*	24,065	17,375	3,945	2053	9	47	2000^30^
*Typha* sp(p).	68	360	10	38	18	91	250000^45^
*Phragmites* sp(p).	2,941	9,898	635	1,001	26	89	500^46^
*Andropogon* sp(p).	351	2,440	64	171	37	62	100^47^

Note that for taxa with ranges of seed production per plant in the literature, we chose the lowest value. For example, with *Andropogon* sp(p).^47^, we were conservative and chose the lowest seed production for any species in the genus, though number of seeds per plant in other species of the genus can be up to 60,000.

**Figure 2:**
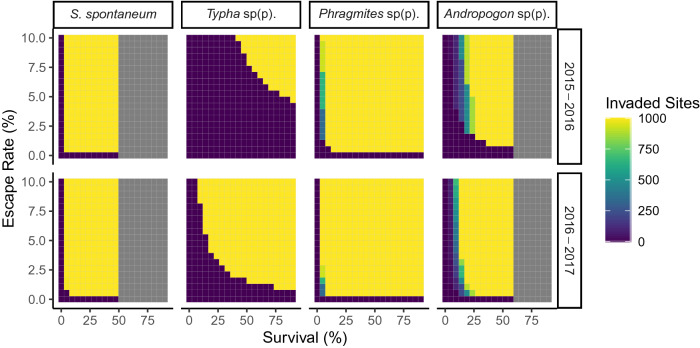
Simulated number of invaded sites after four years. Simulations were based on estimated seed influx in either Season 1 or 2. Invaded sites are those inhabited by at least one reproductive individual. Grey boxes demarcate survival rates above those observed under greenhouse conditions. Created in R version 3.5.1^44^.

Of the 9 taxa that successfully germinated, 5 taxa (55%) survived transplantation to 4″ pots (our measure of survivorship). Of our four focal taxa, *S. spontaneum*, had 47% survivorship (75 of 160 seedlings), *Typha* sp(p). had 91% survivorship (149 of 164 seedlings), *Phragmites* sp(p). had 89% survivorship (160 of 180 seedlings), and *Andropogon* sp(p). had 62% survivorship (39 of 63 seedlings) (Table 1). These substantial survivorship percentages were included in our simulation models below; however, we recognize that ideal laboratory conditions may result in an overestimate of survivorship as compared with field conditions, so we used these values as upper bounds in our modeling.

### Propagule pressure simulation model

**Figure 3:**
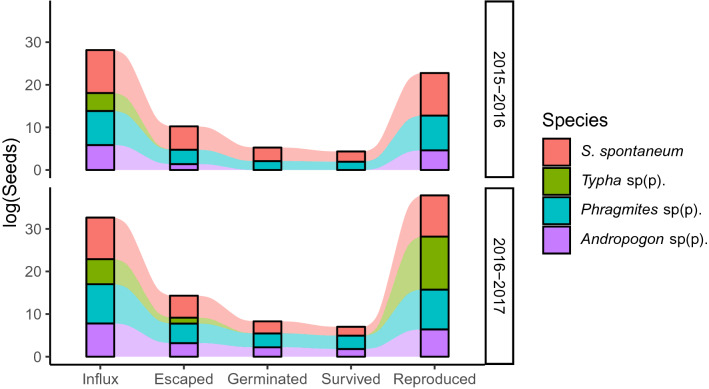
Alluvial figures of model output based on a 1% seed escape rate from refrigerated shipping containers. Influx values are derived from the season of seed sampling (Season 1 = 2015–2016; Season 2 = 2016–2017). Germinated and survived proportions for both seasons were derived from rates calculated from germination and survivorship trials on seeds from Season 2 (see Methods). Reproduced proportions are based on reproductive output of seed estimated from the literature (Table 1). Created in R version 3.5.1^44^.

Invasion risk varied considerably according to estimated total influx (arrivals on shipping containers) and rate of escape (dispersal from shipping containers). For *Phragmites* sp(p). and *S. spontaneum*, we calculated on the order of thousands (*Phragmites*) or tens of thousands (*S. spontaneum*) of seeds having entered the Port of Savannah in each season (Table 1). Even with as few as 0.5% of seeds escaping to a suitable site and survivorship as low as 10%, simulated populations rapidly reached carrying capacity (Fig. 2; Supplementary Fig. 3). In contrast, we estimated the total influx of *Typha* sp(p). at only 68 seeds (Season 1) and 360 seeds (Season 2) (Table 1), and relatively higher escape and survival rates were required for import on shipping containers to pose an invasion risk in our simulations (Fig. 2). Seed influx for *Andropogon* sp(p). varied between seasons, with successful population establishment more likely due to seed influx on shipping containers during Season 2 (Fig. 2 and Supplementary Fig. 3). Notably, whether or not a population became fully established was insensitive to the number of sites available (1,000 or 100), although populations become fully established more quickly at lower carrying capacities. While these models do not take into account processes such as competition, all four focal taxa have such high average reproductive rates that establishment could reasonably be driven by reproduction of even just a few individuals escaping in a single season (Fig. 3).

## Discussion

Changes in propagule pressure from single or multiple regions directly contribute to the success or failure of nonnative species establishment^6,8,23,24^. In this study, we collected and measured the quantity and diversity of seeds, over time obtained from the air-intake grilles of refrigerated containers, with two seasons for comparison. Targeting the trans-oceanic transport of a single commodity in this industrial trade system that serves as a transport vector for hitchhiking seeds provided reduced variation in which to quantify propagule pressure, including propagule size (Fig. 1) and propagule number (Supplementary Fig. 2) of plant species considered to be of high-risk to agriculture in the USA^19^. Our key finding is that influx is sufficient and reproduction of these species is high enough to represent a risk of population(s) establishment in and around the shipping port, even with the bottlenecks of escape from the shipping container, subsequent germination, and seedling survival (Figs. 2, 3).

Over 20,000 shipping containers are moved as import or export daily on the GCT ^14^, providing ample volumes for passive hitchhikers to establish at the GCT and surrounding areas. In fact, we found steady arrival of shipping containers over the approximately 32-week shipping season (Supplementary Fig. 2). Conversely, we found strong seasonal variation in propagule number (i.e., number of seeds per refrigerated shipping container; Fig. 1). We estimated for the FNW, *S. spontaneum*, that over 40,000 seeds entered GCT during the two shipping seasons (Table 1). This level of propagule pressure is clearly sufficient to represent introduction and establishment risk of a clonal, perennial, fecund species that likely does not require a large initial propagule size, even if the escape rate from the shipping containers is exceedingly small (Table 1; Fig. 2). In this study, the four focal monocotyledonous taxa all had similar seed sizes, and no other larger-sized propagative material of these species (e.g., rhizomatous material or cuttings) were encountered during our study at the GCT.

The theoretical literature postulates that increased numbers of propagules (i.e., propagule size^6,8–11^) and pressure (which includes propagule size and frequency as a rate) increases the likelihood of nascent population establishment and population size and diversity^6,10^; however, among our four focal taxa, a nascent population may establish from a single seed during arrival at a suitable terrestrial substrate, such as the GCT’s greenspaces^19^. Persistence of an extremely small population can, and is likely to, be facilitated by asexual propagation and spatial spread of these particular plant taxa. Theoretical population biology intrinsically includes propagule pressure within the invasion process^6,10^, and empirical studies measuring propagule pressure have demonstrated its importance as the most important and generalizable predictor of nonnative invasion success^24^. Propagule pressure in itself is also the factor most influenced by human activity^8,9^. Therefore, our study adds additional support to the importance of propagule pressure (see Figs. 2 and 3), and in this system, there is sufficient propagule pressure (i.e., influx from Fig. 3) for invasion success, even if escape rates from shipping containers, germination, and survival are low.

Though *S. spontaneum* is the only FNW we encountered, we were also able to identify *Arundo donax* L., a species that is listed as noxious by 46 of the USA’s 50 states^25^. We were not able to identify seeds to a taxonomic level sufficient to determine origin status for the other 28 taxa encountered (Table S4), but for our three additional focal species, we suggest that two species are native and one is likely introduced. We found *Typha domingensis* (Pers.) Steud. (native), *Andropogon glomeratus* (Walter) Britton, Sterns & Poggenb. and *A. virginicus* L. (both native), and *Phragmites australis* (Cav.) Steud. (nonnative), already established on-port at the GCT in a previous study that demonstrated that the Port of Savannah is a hub of nonnative species richness^19^.

For any of the species collected on the shipping containers, the propagules have the potential of being picked up *en route* to the GCT or, with the exception of *S. spontaneum* since it is not established there, at the GCT. Most of the taxa already have cosmopolitan distributions, and actual escape rates from the shipping containers are not yet known, meaning that the seeds could make multiple journeys on cargo ships across oceans before being released from a container. Also, the seasonality of seed dispersal coincides with dispersal time in the northern hemisphere, which may apply to seed sources in Panama, the Caribbean, or the USA. *Andropogon glomeratus* and *A. virginicus* occur throughout North and Central America (including Panama and the Caribbean)^26^. As native species to the southeastern USA, propagules escaping refrigerated shipping containers are not of significant concern, although they could be homogenizing genetic composition if genotypes from other portions of the parental established ranges are introduced here. Additionally, *Andropogon* propagules may result in introductions of nonnative species to South America if the seeds remain on the containers and are viable for return trips. *Typha domingensis* has nearly a global distribution, and though it is native to the southeastern USA, its presence at the Port of Savannah could also indicate the presence of admixed genotypes. Moreover, our morphological identification of the seeds could not distinguish to the species level, and *T. angustifolia* L., a nonnative species, could have been represented in our samples, though this species is well established and widely distributed already in the USA^26^. *Phragmites australis*, a noxious weed in 6 USA states^25^, is already found worldwide^26^. The genus *Phragmites* contains 4 species, of which only *Phragmites australis* is native to portions of North America; however, intra- and inter-specific hybridization among genotypes has resulted in the influx of nonnative lineages from Europe and Asia, which have spread to areas of the continent where it is not native^27,28^.

The most interesting case is *S. spontaneum*, the FNW. This species is established only in Florida on the USA mainland, where it was introduced for historical and extant breeding programs with sugarcane^29^. This recent report^29^ showed that it was naturalized in only three counties, but we have documented it growing in six counties and in cultivation in one additional county (Supplementary Fig. 4). We did not find it growing at the GCT at the Port of Savannah. Yet, it is known that *S. spontaneum*, which is native to the Indian subcontinent, is well established in the Panama Canal region^29,30^ along the shipping route of interest. The number of propagules we intercepted and estimated, along with nontrivial germination rates and high survivorship of seedlings, indicate that this species represents a real threat of establishment outside of Florida. Combined with other modeled estimates that *S. spontaneum* can establish throughout the majority of the USA^29^, we suggest that this species represents a significant risk of negative invasive species impact, earning its FNW listing in the 1980s^18,29^.

All four of our focal taxa share common life history features that have been suggested to be characteristic of invasive plant species: asexual reproduction through rhizomes, persistence in a wide range of environmental conditions, prolific seed production (Table 1 and citations within), wind pollination, and wind dispersal^31–33^. These traits have the potential to enhance geographic spread into new ranges and rapidly lead to single-species domination of local plant communities. All of these taxa have a life history and ecology similar to the very successful southeastern USA invasive species cogongrass (*Imperata cylindrica* (L.) P. Beauv.) that has been demonstrated to benefit from intraspecific heterosis and multiple introductions^34–36^.

A previous study used molecular barcoding of seedlings germinated from seed collected from Season 1 in this study, and they identified some seedlings as: *S. spontaneum, Typha* sp(p)., *Phragmites* sp(p)., and *Andropogon* sp(p).^37^, as identified here. Seeds that were grouped as *S. spontaneum* in this study resulted in seedlings that returned haplotypes for the genus *Phragmites* (*rbcL* haplotype 1 and *matK* haplotypes 3 and 4^37^) and *Saccharum* along with other genera^37^. There are two interesting and opposing forces at play here. First, in sorting seeds morphologically, there is the potential to group similar looking seeds of different species. The molecular barcode result that shows *Phragmites* haplotypes in seeds morphologically identified as *S. spontaneum* is evidence of misidentification and inaccurate sorting of seed. Second, some haplotypes showed equally correct molecular identification across multiple genera of grasses, indicating that these standard molecular barcode sequences for plants may not have the species-level resolution necessary for molecular identification of some of the highest threat invasive grass species.

There are two key approaches to mitigating the risk that propagules of nonnative taxa will become established: 1) prevent propagules from hitchhiking on transoceanic cargo ships, in this case, becoming attached to shipping containers at their point-of-origin or stops along the way (that result from “trans-shipping”), and 2) prevent viable propagules from entering and establishing in the USA, via inspection and interception by the “gatekeepers” of biosecurity at international points-of-entry. These agricultural inspectors are tasked with the interception of propagules of insects, fungi, and all other nonnative or “actionable” taxa, in addition to the seeds of plants. One potential solution to reduce invasion risk by vascular plant seed is to employ a scaled-up version of the research approach we implemented here of backpack vacuuming air-intake grilles of refrigerated shipping containers. Another possibility in lieu of labour-intensive vacuuming of intake grilles is to conduct research on efficacy of liquid pre-emergent herbicide application to the air-intake grilles. For either approach, our data support that these interventions may not be needed year-round for important species like *S. spontaneum*, which have a clear import seasonality on this particular commodity. For example, based on our data, seed removal measures may only be needed in October, November, and early-mid December.

In the face of poorly resourced capacity for inspection and the potential of diminishing fiscal resources and human capital, consequences include acceleration of biodiversity loss, economic and environmental impacts, and on-going biotic homogenization. The interception efforts to prevent the entry of nonnative propagules of all nonnative taxa worldwide will ultimately conserve local endemism, biodiversity, economic output, and ecosystem services that are interrupted or extirpated by biological invasions^1,3^. This research aimed to identify key risks and highlights the need for improved strategies for efficacious prevention and interception of nonnative, particularly plant, propagules prior to establishment, though such prevention approaches can be designed and applied for many taxa. Enhancing the capacity, speed, and frequency of successful prevention programs will be required to minimize or eliminate the real risks posed by viable hitchhiking propagules associated with economic trade and sea/air transportation of commodities and people.

## Methods

### Study site

The Garden City Terminal (GCT) is the container-handling facility of the Port of Savannah and is located in Chatham County, Georgia, USA (32°07′42.5"N, 81°09′05.4"W) (Supplementary Fig. 1; also see: Lucardi et al.^19^).

### Sample collection

We focused on seed collection from the air-intake grilles of refrigerated shipping containers, and therefore, limited our “targets” to arrivals of a single agricultural food commodity that we have anonymized to protect private commerce and customer information per our agreement with the Georgia Port Authority (GPA). Refrigerated shipping containers at the GCT are stacked four containers tall (stories 1–4) and five deep (location A-E) in an innovative footprint-saving approach led and implemented by the GPA. These refrigerated container racks are open-air metal stairs and platforms for easy access to power for the refrigerated shipping containers. This design allows for safe and efficient access for the longshoremen who connect and disconnect the containers from electrical power and the gantry operators who move containers onto drays or over-the-road trucks for commodity distribution off-port.

These refrigerated container racks were constructed just shortly before this research was initiated, which allowed the research team to access as many targeted containers as possible. For collection of seeds attached to the outside of air-intake grilles, we utilized consumer grade, 110 V backpack vacuums with plastic tube attachments. A synthetic textile sample collection bag was inserted into the vacuum tube and held in place with the plastic angled vacuum attachment. Each refrigerated container was sampled into a separate synthetic textile bag (i.e., a nylon/spandex blend with low porosity that allows for high air exchange; dimensions fit the plastic tube extenders ideally and were not > 30 cm in length) for collection of all vacuumed debris. These textile bags are easily accessible and were new, ladies’ trouser socks. Collection textile bags were placed into individual labelled plastic bags with date of collection, container identification code, and the initials of the sampling researcher. All individual plastic bags were then placed into a gallon-sized plastic bag with the date of collection for downstream sorting processes. The research team was always escorted by a USCBP Agriculture Supervisor while on-port for safety and on-the-fly technical support.

Prior to arrival on-port for seed collection, the research team coordinated with the Client Relations Center of GPA to receive weekly reports of ship arrivals and the number of containers of the agricultural commodity. Weather events, including tropical cyclones, could delay ships and therefore required the research team to remain nimble to arrive on-port when the greatest number of target containers would be present. The morning of each sampling day, the Client Relations Center provided us exact locations of our target containers on-port at the GCT. On occasion, some of our target containers were moved onto over-the-road trucks as we arrived at the rack to sample them, so speed and flexibility were required in accessing and obtaining the highest number of samples possible for each sampling day.

The shipping season of interest in this study generally occurs from August through February. We initiated the sampling protocol on 13 August 2015 for the 2015–2016 season (Season 1) and completed sampling on 1 February 2016. The sampling protocol did not change for Season 2, which encompassed sampling dates from 15 August 2016 to 27 February 2016. Also in Season 2, we conducted germination and post-transplant survivorship protocols on sampled seeds (see below). All refrigerated shipping container arrivals, whether we sampled them or not, were recorded so we were able to compare our number of assessed containers with arrivals (Tables S1 and S2). For our target commodity, Season 1 resulted in 1858 total 40-ft (2 Twenty-foot Equivalent container Units [TEU]) refrigerated shipping container arrivals and Season 2 resulted in 1925 (Supplementary Fig. 2; Table S1 in Supplementary Information). Of those, 331 refrigerated shipping containers (18%) were sampled in Season 1 during 15 on-port sampling visits, and 297 containers (15%) were sampled in Season 2 during 14 visits (Supplementary Fig. 2; Table S2).

### Seed sorting

Rough morphological sorting of seeds was first conducted for each container sample bag and sorted by eye to remove seed-like structures from other debris, such as insect sections, litter, and hair. The sorting of seeds into different morphotypes and the counting of the number of seeds in each morphotype was conducted under a stereo-microscope with an on-board camera (AmScope, 14,730 Myford Road #150, Irvine, CA 92,606, USA; Table S3). Seeds were sorted into 22 morphotypes for germination trials (Table S4). Examples of morphotypes were photographed. Seed vouchers (a maximum of 22 seeds per morphotype) and morphotype photographs were sent to the Arkansas State University Herbarium (STAR) for identification of all morphotypes. Seeds were identified at STAR using descriptions and image comparisons from a variety of keys (see^38–41^) and sources including online image comparisons from a variety of websites under a Fisher Stereomaster dissecting microscope. Sixty samples were accessioned into the seed collection at STAR for permanent vouchering of specimens from this research (STAR035370-STAR035429).

### Estimating total influx of seeds

We estimated the total influx of seeds in Seasons 1 and 2 based on seed collection events that took place approximately once every two weeks. We focused on four monocotyledonous taxa (identified to species or genus level) with the highest number of seeds collected in Season 2. The distribution of seed counts over the season varied considerably for each species and year, often with multiple peaks of different sizes during the season. Correspondingly, we used *K*-Nearest Neighbours (KNN) regression to estimate seeds per container based on ship arrival date. KNN regression is a non-parametric statistical learning approach that estimates the response value for an unknown observation as the average of the *K* nearest observations from the training dataset^42^. We trained separate KNN regression models for each season and species, and we estimated the seeds per container on a given ship arrival date based on the average of the *K* = 2 nearest sampling dates. Total influx of seeds for each season was calculated as $N=\sum_{i=1}^{t}{s}_{i}\times {c}_{i}$, where N is the total influx of seeds, *s*_*i*_ is the predicted number of seeds per container on arrival date *i*, and *c*_*i*_ is the number of containers on arrival date *i*.

### Germination trials

For each seed taxon (Table S2), trials were conducted to quantify germination success and survivability of seedlings. Seeds were already sorted (see above), and to ensure that germination trials would be accurate, seed coats and other dispersal accessories were removed with tweezers under a stereo-microscope (AmScope). Naked, fully formed seeds with obvious, fully intact endosperm were utilized for germination trials.

Trials were conducted by placing 10 seeds of the same taxon onto filter paper in plastic petri dishes. If quantities allowed, each plate contained seeds from one shipping container. For those with fewer numbers, container identifiers were pooled to make 10 seeds of the same taxon per germination dish. Each dish was individually labelled and dated. The duration of trials lasted 14 days in a germination chamber (Nor-Lake Scientific, Hudson, WI, USA). Throughout all trials, the chamber maintained a 12/12-h light cycle, with temperatures set at 29 °C during the light and 20 °C during the dark. Dishes were provided RO water via serological pipettes.

Germination dishes were checked and recorded daily during the 14-day trial. We selected a 14-day interval to handle the volume of seeds as well as petri dishes due to space limitations in the germination chamber. Absence of germination by seeds after 14 days tended to result in rotted seeds in the petri dish that contributed to mold formation that could impact other seeds in the same dish or contaminate other dishes undergoing germination trials in the chamber simultaneously. After the 14-day trial, the number of germinated seedlings per dish were counted, and a percent germination was calculated based on the original 10 seeds in the dish. Surviving seedlings after the 14-day trial were transplanted into a 72-cell seedling tray with Sunshine Mix 1 (Sun Gro Horticulture, Agawam, MA, USA) and a ¼ teaspoon of Osmocote (The Scotts Company, Marysville, OH, USA), watered with tap water until saturated, and returned to the germination chamber for continued growth.

After 14 days of daily checks, dishes with no seedlings or no remaining seedlings due to transplant were destroyed in the autoclave, in accordance with our USDA APHIS FNW permit. In some cases, seedlings remained in the dish even after the 14-day trial, to develop further before transplant; these dishes were allowed 3–4 more days (maximum) before being destroyed. In this manner, the already recorded seedlings in the dish would not be recorded in duplicate or lost due to the assigned 14-day germination period. Plants in the 72-cell tray were watered daily (or as needed to avoid overwatering), and a clear tray-cap was kept on the tray in the germination chamber to increase humidity for the seedlings. Plants that outgrew the 72-cell tray by becoming rootbound, were transplanted to individual 4″ standard plastic pots, flagged, and tagged. This was the stage at which we measured percent survival based on the number germinated. Those seedlings that outgrew 4″ pots were transplanted to 1-gal standard plastic pots and assigned a metal tag and were transported in a closed vehicle to a quarantine greenhouse facility in Athens, GA (USFS; USDA APHIS PPQ 526 P526-150,805–006 to TDM and P526-190,626–009 to RDL), at the USFS property at the University of Georgia in Athens.

### Simulating establishment of invasive populations

To evaluate whether seeds hitchhiking on air-intake grilles could lead to establishment of new populations of non-native or FNW species, we simulated establishment in greenspaces of the GCT using a process-based demographic model^20^. Because the total influx of seeds differed across years (see Results), we considered the impact of propagules coming in during a single season and simulated population dynamics over 10 years. Each year, we recorded change in the number of ‘invaded sites’, which we defined as a 1-m^2^ area inhabited by at least one reproductive individual of the species under consideration. The GCT in total is greater than 485 ha and interspersed with small greenspaces throughout^19^. We asked if one area of 250m by 4m (equivalent to 1,000 available sites) could become fully occupied within several years. A 1,000-m^2^ area represents approximately 15% of an average greenspace at the GCT^19^ and is similar to the median minimum viable population size required for persistence over ~ 100 years estimated from 1,200 species^43^. Smaller populations may be able to persist over shorter timescales, and we also investigated lower values (e.g., 100 available sites). Because seeds from all species under consideration could potentially disperse from a single reproducing individual within a greenspace of about this size, simulations were performed under the ‘mean field’ hypothesis, which assumes that each available site is equally susceptible to establishment^20^. Consequently, our model does not take into account variation in the probability of establishment among 1-m^2^ sites, which could be caused, for example, by competition with already established plant communities.

We modeled four processes, each of which acted multiplicatively to reduce the number of seeds that eventually survived to become a reproductive adult: escape of seeds from shipping containers, seed germination, seedling survival, and reproduction of established individuals. In the first year of the simulation, the number of reproductive individuals depended solely on influx of seeds from shipping containers (Table 1) or the ‘escape rate’, defined as the proportion of seeds coming in from shipping containers that landed on a suitable site. Because the actual escape rate is unknown, we considered a range of possible values between 0.01% and 10%. The number of sites with an escaped seed was further modified by the germination rate to take into account differences in germination success among species (Table 1). After germination, seedlings survived according to the survival rate; we considered survivorship estimated by those seedlings that survived to transplant to 4″ pots as an upper bound for each species (Table 1), but also simulated lower survival rates to account for increased mortality outside of the controlled conditions in this research. After one year of vegetative growth, all surviving individuals contributed propagules to the next generation (e.g., through sexual reproduction) and remained established for the duration of the simulation (e.g., through vegetative reproduction from rhizomes) for our focal taxa. After the first year, seed influx at the beginning of the year depended only on reproduction by established individuals using plant reproduction values obtained from the literature (see Table 1). All simulations were implemented in R version 3.5.1^44^; code to run the simulations is provided in the Supplementary Information.

## Supplementary information

Supplementary Tables

Supplementary Methods

Supplementary Figures

## Data Availability

All data and code are available in the Supplementary Information Tables 1–5 and the Supplementary Methods.
