# Supplementary material for: Seeds attached to refrigerated shipping containers represent a substantial risk of nonnative plant species introduction and establishment
Source: Sci Rep. 2020 Sep 14;10:15017. doi: 10.1038/s41598-020-71954-3 (PMC7490705; doi:10.1038/s41598-020-71954-3)
Supplement: Supplementary file 2 — Supplementary Methods [file 41598_2020_71954_MOESM6_ESM.docx]

**Title**: Seeds attached to refrigerated shipping containers represent a substantial risk of nonnative plant species introduction and establishment

**Authors**: Rima D. Lucardi^1*^, Emily S. Bellis^2^, Chelsea E. Cunard^3^, Jarron K. Gravesande^3#^, Steven C. Hughes^4^, Lauren E. Whitehurst^5#^, Samantha J. Worthy^5#^, Kevin S. Burgess^5^, Travis D. Marsico^3*^

^1^United States Dept. of Agriculture, Forest Service, Southern Research Station, Athens, GA. ^2^Arkansas Bioscience Institute and Dept. of Computer Science, Arkansas State University, Jonesboro, AR. ^3^Dept. of Biological Sciences, Arkansas State University, Jonesboro, AR. ^4^The Herbarium at the University of Georgia, Dept. of Plant Biology, Athens, GA. ^5^Dept. of Biology, Columbus State University

***Corresponding authors**: Rima D. Lucardi, [rima.lucardi@usda.gov](mailto:rima.lucardi@usda.gov); Travis D. Marsico, [tmarsico@astate.edu](mailto:tmarsico@astate.edu)

^#^**Present address**: JKG Department of Plant Pathology, University of Georgia, Athens, GA, USA; LEW Department of Biology, University of Florida, Gainesville, FL, USA; SJW Department of Biology, University of Maryland, College Park, Maryland, USA.

**ORCID**: RDL (0000-0002-8851-2494), SJW (0000-0003-0414-2607), LEW (0000-0002-3317-0028), ESB (0000-0001-6066-1466), TDM (0000-0002-8422-8314)

Supplemental Methods

R code to recreate analyses and figures are presented here.

**Ship Arrivals.** This code plots the number of containers and proportion of containers sampled per week in each season.

library(ggplot2)
library(dplyr)
library(lubridate)
library(tidyr)
library(reshape2)

## format and plot data for incoming ships
ships <- read.csv('port_containers_20200312.csv', header=T)
ships$date <- as.Date(ships$date, format="%m/%d/%Y")

ships2 <- subset(ships, season==2) %>% group_by( ym = week(date)+(53*(year(date)-0016)))
ships1 <- subset(ships, season==1) %>% group_by( ym = week(date)+(53*(year(date)-0015)))
ships <- rbind.data.frame(ships1, ships2)

sampled <- read.csv('containers_sampled_20200312.csv', header=T)
sampled$date <- as.Date(sampled$date, format="%m/%d/%Y")
sampled2 <- subset(sampled, season==2) %>% group_by( ym = week(date)+(53*(year(date)-0016)))
sampled1 <- subset(sampled, season==1) %>% group_by( ym = week(date)+(53*(year(date)-0015)))
sampled <- rbind.data.frame(sampled1, sampled2) %>% select(season, ym, containers_sampled) %>% group_by(season,ym) %>% summarize(cont_sampled= sum(containers_sampled))

## Figure S2:
seasons <- c(
 "1" = "2015-2016",
 "2" = "2016-2017"
)

tmp.4 <- ships %>% group_by(season, ym) %>% summarize(cont = sum(containers))
tmp.5 <- left_join(tmp.4, sampled) %>% select(season, ym, cont, cont_sampled)
tmp.5$cont_sampled[is.na(tmp.5$cont_sampled)] <- 0
tmp.5$unsampled <- tmp.5$cont-tmp.5$cont_sampled
tmp.5$unsampled[tmp.5$unsampled < 0] <- 0
tmp.6 <- pivot_longer(tmp.5, c(cont_sampled, unsampled))

pdf(file="FigS2-v2-ESB.pdf", height=3, width=6)
ggplot(tmp.6, aes(x=ym, y=value, fill=name, group=season)) + geom_col(col="black", lwd=0.25) + facet_grid(season~., labeller = labeller(season= seasons)) + theme_classic() + xlab("Week") + ylab("Containers Arriving")+ scale_x_continuous(breaks=c(30,40,50,60), labels=c("30","40","50","7")) + scale_fill_manual(name="", values=c('grey15','grey80'), labels=c("Sampled","Not sampled"))
dev.off()

**Estimating Seed Influx.** We estimated the total influx of seeds in each season based on seed collection events that took place approximately once every two weeks. A KNN regression model was trained on data from each season to predict the number of seeds per container for four seed types based on ship arrival date and the average estimate from the *K*=2 closest sampling dates. The total influx was calculated as the sum of the predicted number of seeds per container on a particular arrival date, multiplied by the number of containers containing the commodity, for ships entering the Port of Savannah during the season.

library(caret)
## format data for seeds sampled
seeds <- read.csv('containers_sampled_20200312.csv', header=T)
seeds$date <- as.Date(seeds$date, format="%m/%d/%Y")
seeds$jwk <- week(seeds$date)
seeds2 <- subset(seeds, season==2) %>% group_by(ym = week(date)+(53*(year(date)-0016)))
seeds1 <- subset(seeds, season==1) %>% group_by(ym = week(date)+(53*(year(date)-0015)))
seeds <- rbind.data.frame(seeds1, seeds2)
seeds.long <- melt(seeds, id=c("containers_sampled", "date","ym","jwk","season"))
seeds$per_1 <- seeds$type_1/seeds$containers_sampled
seeds$per_5 <- seeds$type_5/seeds$containers_sampled
seeds$per_8 <- seeds$type_8/seeds$containers_sampled
seeds$per_10 <- seeds$type_10/seeds$containers_sampled

SeedType <- c(1,5,8,10)

## fit models
for (i in 1:2) {
 TotalSeeds <- NULL
 curr.seeds <- subset(seeds, season==i)
 curr.ships <- subset(ships, season==i)

 knn.1.fit <- knnreg(per_1 ~ ym, data=curr.seeds, k=2)
 knn1.df <- rbind.data.frame(cbind.data.frame(id="observed",type="type_1",ym=curr.seeds$ym, nseeds=curr.seeds$per_1),cbind.data.frame(id="KNN (K=2)",type="type_1",ym=curr.ships$ym, nseeds=predict(knn.1.fit, curr.ships)))
 TotalSeeds <- c(TotalSeeds,round(sum(curr.ships$containers * predict(knn.1.fit, curr.ships))))

 knn.5.fit <- knnreg(per_5 ~ ym, data=curr.seeds, k=2)
 knn5.df <- rbind.data.frame(cbind.data.frame(id="observed",type="type_5",ym=curr.seeds$ym, nseeds=curr.seeds$per_5),cbind.data.frame(id="KNN (K=2)",type="type_5",ym=curr.ships$ym, nseeds=predict(knn.5.fit, curr.ships)))
 TotalSeeds <- c(TotalSeeds, round(sum(curr.ships$containers * predict(knn.5.fit, curr.ships))))

 knn.8.fit <- knnreg(per_8 ~ ym, data=curr.seeds, k=2)
 knn8.df <- rbind.data.frame(cbind.data.frame(id="observed",type="type_8",ym=curr.seeds$ym, nseeds=curr.seeds$per_8),cbind.data.frame(id="KNN (K=2)",type="type_8",ym=curr.ships$ym, nseeds=predict(knn.8.fit, curr.ships)))
 TotalSeeds <- c(TotalSeeds,round(sum(curr.ships$containers * predict(knn.8.fit, curr.ships))))

 knn.10.fit <- knnreg(per_10 ~ ym, data=curr.seeds, k=2)
 knn10.df <- rbind.data.frame(cbind.data.frame(id="observed",type="type_10",ym=curr.seeds$ym, nseeds=curr.seeds$per_10),cbind.data.frame(id="KNN (K=2)",type="type_10",ym=curr.ships$ym, nseeds=predict(knn.10.fit, curr.ships)))
 TotalSeeds <- c(TotalSeeds, round(sum(curr.ships$containers * predict(knn.10.fit, curr.ships))))

 assign(paste0("TotalSeeds_Yr",i),TotalSeeds)
 assign(paste0("ObsSeeds_Yr",i), c(sum(curr.seeds$type_1), sum(curr.seeds$type_5),sum(curr.seeds$type_8),sum(curr.seeds$type_10)))

 tmp <- rbind.data.frame(knn1.df, knn5.df, knn8.df, knn10.df)
 tmp$yr <- i
 assign(paste0("tmp",i),tmp)
}

tmp <- rbind.data.frame(tmp1, tmp2)

tmp$type <- factor(tmp$type, labels = c('italic("S. spontaneum")','italic("Typha")~"\nsp(p)."','italic("Phragmites")~"sp(p)."','italic("Andropogon")~"\nsp(p)."'))

tmp$yr <- factor(tmp$yr, labels=c("2015-2016","2016-2017"))

## Figure 1
q <- ggplot(tmp, aes(x=ym, y=nseeds, col=id, lty=id)) + geom_line() + geom_point(size=0.6, alpha=0.7)+facet_grid(yr~type, labeller=label_parsed) + theme_classic() + scale_colour_manual(values=c("grey","black"),name="") + xlab("Week") + ylab("Seeds per Container")+scale_linetype_discrete(name="") + scale_x_continuous(breaks=c(30,40,50,60), labels=c("30","40","50","7"))+theme(strip.text.x = element_text(face = "italic"))

pdf(file="Fig1-v1-ESB.pdf", height=3.5, width=7)
q
dev.off()

perYr <- cbind.data.frame(SeedType, TotalSeeds_Yr1, TotalSeeds_Yr2, ObsSeeds_Yr1, ObsSeeds_Yr2)

**Simulating establishment of invasive populations.** We developed a process-based simulation model to predict the potential success of newly established invasive populations based on observed propagule pressure at the Port of Savannah (see Methods in manuscript text).

library(wesanderson)

perYr$Germ <- c(0.10, 0.18, 0.26, 0.37)
perYr$Surv <- c(0.47, 0.91, 0.89, 0.62)
perYr$Repr <- c(2000, 250000, 500, 100)

df <- data.frame(Species = factor(), Escape = numeric(), Year = integer(), Seeded = numeric(), Germinated = numeric(), Reproductive = numeric(), Season = numeric(), stringsAsFactors=FALSE)

df.both <- NULL

for (y in 1:2) {#y iterates over years

for (s in 1:4) { #s iterates over species

###########initial params
Year = 0
CC = 1000 #carrying capacity
SeedS = 0
GermS = 0
ReproS = 0
CORF = 1 #correction factor for proportion of suitable sites still available for introduction

# Rates/coefficients:
GERMINATION = perYr$Germ[s]
SURVIVAL = perYr$Surv[s]
REPRODUCTION = perYr$Repr[s]
ESCAPE = c(0.0001, 0.001, 0.01, 0.10)

##############run the simulation
for (e in 1:4) { #e iterates over escape rates
 for (i in 1:10) {#i iterates over years, assume year starts in April 2017
 if(i == 1 && y == 1){INFLUX = perYr$TotalSeeds_Yr1[s]} else if(i == 1 && y == 2){INFLUX = perYr$TotalSeeds_Yr2[s]} else {INFLUX = 0} #only look at effect of seed influx from a single year
 if(i == 1){ReproS=0} #assume no reproduction in first year
 SeedS = CORF*((INFLUX*ESCAPE[e]) + (ReproS*REPRODUCTION))
 GermS = SeedS*GERMINATION
 ReproS = round(min((GermS*SURVIVAL + ReproS), CC))
 CORF = 1 - (ReproS/(CC))

 df <- rbind(df, cbind.data.frame(Species = SeedType[s], Escape = ESCAPE[e], Year = i, Seeded = SeedS, Germinated = GermS, Reproductive = ReproS, Season = y))
 }
Year = 0
SeedS = 0
GermS = 0
ReproS = 0
CORF=1
}
}
 df.both <- rbind.data.frame(df.both,df)
}

df.both$Species <- factor(df.both$Species, labels = c('italic("S. spontaneum")','italic("Typha")~"\nsp(p)."','italic("Phragmites")~"sp(p)."','italic("Andropogon")~"\nsp(p)."'))

df.both$Season <- factor(df.both$Season, labels=c("2015-2016","2016-2017"))

## Figure S3
p <- ggplot(df.both, aes(x=Year, y=Reproductive/CC*100, col=as.factor(Escape), lty=as.factor(Escape), group=Escape)) + geom_line(size=1) + ylab("Invaded Sites (%)") + theme_classic() + scale_linetype_discrete(name="Escape Rate")+ scale_color_manual(name="Escape Rate", values=wes_palette("Zissou1", 4, "continuous")) + facet_grid(Season~Species, labeller=label_parsed) + scale_x_continuous(breaks=c(0,2,4,6,8,10))+theme(strip.text.x = element_text(face = "italic"))

pdf(file="FigS3-v2-ESB.pdf", width=7, height=3.5)
p

dev.off()

**Part IIb: Simulating establishment with varying survival & escape rates, with greenhouse-estimated survival as an upper bound.**

*#create a function that calculates number of invaded sites for each species after 4 years given escape, survival, species, and season*
SimYrs <- **function**(escape, survival, species, season) {
 Year = 0
 CC = 1000 *#carrying capacity*
 SeedS = 0
 GermS = 0
 ReproS = 0
 CORF = 1 *#correction factor for proportion of suitable sites still available for introduction*

 *#Rates/coefficients:*
 GERMINATION = perYr**$**Germ[species]
 REPRODUCTION = perYr**$**Repr[species]
 ESCAPE = escape
 SURVIVAL = survival

 **for** (i **in** 1**:**4) {*#i iterates over years, assume year starts in April*
   **if**(i **==** 1 **&&** season **==** 1){INFLUX = perYr**$**TotalSeeds_Yr1[species]} **else** **if**(i **==** 1 **&&**season **==** 2){INFLUX = perYr**$**TotalSeeds_Yr2[species]} **else** {INFLUX = 0}
   **if**(i **==** 1){ReproS=0} *#assume no reproduction in first year*
   SeedS = CORF*****((INFLUX*****ESCAPE) **+** (ReproS*****REPRODUCTION))
   GermS = SeedS*****GERMINATION
   ReproS = **round**(**min**((GermS*****SURVIVAL **+** ReproS), CC))
   CORF = 1 **-** (ReproS**/**(CC))
 }

 df <- **cbind.data.frame**(Species = perYr**$**SeedType[species], Escape = escape, Survival =survival, Reproductive = ReproS, Season = season)
**return**(df)
}

*## create a dataframe for all the escape x survival combinations*
ESCAPE = **seq**(0.0001, 0.1, length.out=20)
SURVIVAL = **seq**(0.0001, 0.9, length.out=20)

df <- **data.frame**(Species = **factor**(), Escape = **numeric**(), Survival = **numeric**(),Reproductive =**numeric**(), Season = **numeric**(), stringsAsFactors=FALSE)

**for** (y **in** 1**:**2) {
 **for** (s **in** 1**:**4){
   **for** (e **in** 1**:length**(ESCAPE)) {
     **for** (v **in** 1**:length**(SURVIVAL)) {
       df <- **rbind**(df, **SimYrs**(ESCAPE[e],SURVIVAL[v],s,y))
     }
   }
 }
}

*## change values outside of upper bound for survival to 'NA'*
df[df**$**Species**==**10 **&** df**$**Survival **>** 0.6,]**$**Reproductive <- "NA"
df[df**$**Species**==**1 **&** df**$**Survival **>** 0.5,]**$**Reproductive <- "NA"
df**$**Reproductive <- **as.numeric**(df**$**Reproductive)

*## plot!*
**library**(viridis)

## Loading required package: viridisLite

df**$**Species <- **factor**(df**$**Species, labels = **c**('italic("S. spontaneum")','italic("Typha")~"\nsp(p)."','italic("Phragmites")~"sp(p)."','italic("Andropogon")~"\nsp(p)."'))

df**$**Season <- **factor**(df**$**Season, labels=**c**("2015-2016","2016-2017"))

p <- **ggplot**(df, **aes**(x=Survival*****100, y=Escape*****100, fill=Reproductive)) **+geom_tile**(size=0.0001, col="grey") **+** **scale_fill_viridis**(name="Invaded Sites",option ="D")

**pdf**(file="Fig2-v2-ESB.pdf", width=7, height=3.5)
p **+** **facet_grid**(Season **~** Species, labeller=label_parsed) **+** **theme_classic**() **+** **xlab**("Survival (%)") **+** **ylab**("Escape Rate (%)" )

**Visualize Processes.** Visualize contribution of each process to invasion risk with alluvial plot.

library(ggalluvial)

## Figure 3
df2 <- cbind.data.frame(Species = c("1","5","8","10"), Influx = perYr$TotalSeeds_Yr2)
df2$Escaped = round(df2$Influx*0.01)
df2$Germinated = round(df2$Escaped*perYr$Germ)
df2$Survived = round(df2$Germinated*perYr$Surv)
df2$Reproduced = df2$Survived*perYr$Repr
df2$Season = "2016-2017"

df1 <- cbind.data.frame(Species = c("1","5","8","10"), Influx = perYr$TotalSeeds_Yr1)
df1$Escaped = round(df1$Influx*0.01)
df1$Germinated = round(df1$Escaped*perYr$Germ)
df1$Survived = round(df1$Germinated*perYr$Surv)
df1$Reproduced = df1$Survived*perYr$Repr
df1$Season = "2015-2016"

df3 <- rbind.data.frame(df1, df2)
df3 <- melt(df3)

## Using Species, Season as id variables

df3$Species <- factor(df3$Species, labels = c('italic("S. spontaneum")','italic("Typha")~"\nsp(p)."','italic("Phragmites")~"sp(p)."','italic("Andropogon")~"\nsp(p)."'))

df3$Season <- factor(df3$Season, labels=c("2015-2016","2016-2017"))

pdf(file="Fig3-v2-ESB.pdf", height=3.5, width=6)
ggplot(df3, aes(x=variable, y=log(value), stratum=Species, fill=Species, alluvium=Species)) + geom_flow() + geom_stratum() + xlab("") + ylab("log(Seeds)") + theme_classic() + scale_fill_discrete(labels=expression(italic("S. spontaneum"),paste(italic("Typha")," sp(p)."),paste(italic("Phragmites")," sp(p)."),paste(italic("Andropogon")," sp(p)."))) + theme(legend.text.align = 0) + facet_grid(Season~.)

dev.off()

Supplementary Methods for Supp. Figures 1 and 4

Georeferenced maps for Supplementary Figures 1 and 4 were generated in QGIS 3.10 ( <https://www.qgis.org/en/site/index.html)> and the map data were obtained from open sources: US borders and waterways: [https://www.census.gov/cgi-bin/geo/shapefiles/index.php](https://gcc02.safelinks.protection.outlook.com/?url=https%3A%2F%2Fwww.census.gov%2Fcgi-bin%2Fgeo%2Fshapefiles%2Findex.php&data=02%7C01%7C%7C21cf8260a71d480e96e308d833b4bb15%7Ced5b36e701ee4ebc867ee03cfa0d4697%7C0%7C0%7C637316198745153238&sdata=nmq6RAaX%2BZ66Nlt6WxC0zE4a4D7noZRup7zEtuIPczE%3D&reserved=0), International borders: [https://gadm.org/](https://gcc02.safelinks.protection.outlook.com/?url=https%3A%2F%2Fgadm.org%2F&data=02%7C01%7C%7C21cf8260a71d480e96e308d833b4bb15%7Ced5b36e701ee4ebc867ee03cfa0d4697%7C0%7C0%7C637316198745153238&sdata=O52JKNTQE1RqEwaC5rZRFKUhy75g70bRa3jbZiTWZH0%3D&reserved=0). The distribution and records of Saccharum spontaneum distribution was downloaded from [http://sernecportal.org/portal/](https://gcc02.safelinks.protection.outlook.com/?url=http%3A%2F%2Fsernecportal.org%2Fportal%2F&data=02%7C01%7C%7C21cf8260a71d480e96e308d833b4bb15%7Ced5b36e701ee4ebc867ee03cfa0d4697%7C0%7C0%7C637316198745153238&sdata=BnOA2mvBUG7Z1iLqaYDjkcqf5ZLACLAZODuYK2NisMY%3D&reserved=0) on 2020-05-06, and annotated (Provided in Supplementary Table 5).
