# Supplementary material for: Seeds attached to refrigerated shipping containers represent a substantial risk of nonnative plant species introduction and establishment
Source: Sci Rep. 2020 Sep 14;10:15017. doi: 10.1038/s41598-020-71954-3 (PMC7490705; doi:10.1038/s41598-020-71954-3)
Supplement: Supplementary file 3 — Supplementary Figures [file 41598_2020_71954_MOESM7_ESM.docx]

**Title**: Seeds attached to refrigerated shipping containers represent a substantial risk of nonnative plant species introduction and establishment

**Authors**: Rima D. Lucardi^1*^, Emily S. Bellis^2^, Chelsea E. Cunard^3^, Jarron K. Gravesande^3#^, Steven C. Hughes^4^, Lauren E. Whitehurst^5#^, Samantha J. Worthy^5#^, Kevin S. Burgess^5^, Travis D. Marsico^3*^

^1^United States Dept. of Agriculture, Forest Service, Southern Research Station, Athens, GA. ^2^Arkansas Bioscience Institute and Dept. of Computer Science, Arkansas State University, Jonesboro, AR. ^3^Dept. of Biological Sciences, Arkansas State University, Jonesboro, AR. ^4^The Herbarium at the University of Georgia, Dept. of Plant Biology, Athens, GA. ^5^Dept. of Biology, Columbus State University

***Corresponding authors**: Rima D. Lucardi, [rima.lucardi@usda.gov](mailto:rima.lucardi@usda.gov); Travis D. Marsico, [tmarsico@astate.edu](mailto:tmarsico@astate.edu)

^#^**Present address**: JKG Department of Plant Pathology, University of Georgia, Athens, GA, USA; LEW Department of Biology, University of Florida, Gainesville, FL, USA; SJW Department of Biology, University of Maryland, College Park, Maryland, USA.

**ORCID**: RDL (0000-0002-8851-2494), SJW (0000-0003-0414-2607), LEW (0000-0002-3317-0028), ESB (0000-0001-6066-1466), TDM (0000-0002-8422-8314)

**Supplementary Figures**


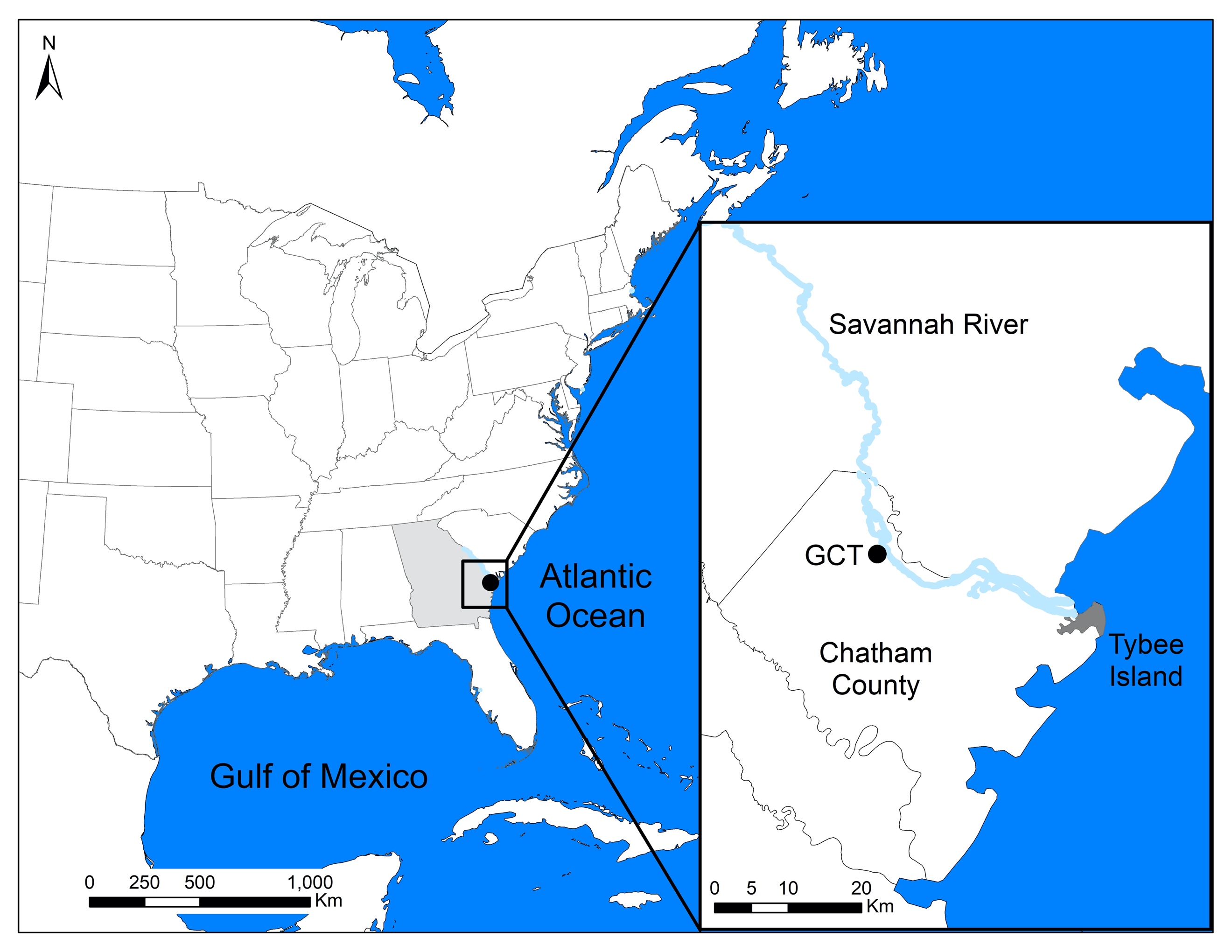


**Supplementary Figure 1.** The Garden City Terminal (GCT) at the Port of Savannah, Georgia, USA. The large map shows the location of the GCT in the southeastern USA, and the inset displays specific location details of the GCT situated along the Savannah River. Map generation in the Supplementary Methods.

**Supplementary Figure 2.** The number of refrigerated shipping container arrivals of our single, agricultural commodity in Seasons 1 and 2, showing total number of arrivals per week in grey and the number of sampled containers of the total arriving in black. Created in R version 3.5.1^44^.

**Supplementary Figure 3.** Process-based simulation model using total estimated seed influx for Seasons 1 and 2. Sites of establishment are shown as the proportion of total available sites (of 1,000) inhabited by a reproducing individual. Simulations are shown over 10 years based on a single influx of seeds in Year 1 with no reproduction in the first year, following the life history of these perennial plant species. For this analysis, empirical germination and survivorship rates (considered maxima) are used. Created in R version 3.5.1^44^.


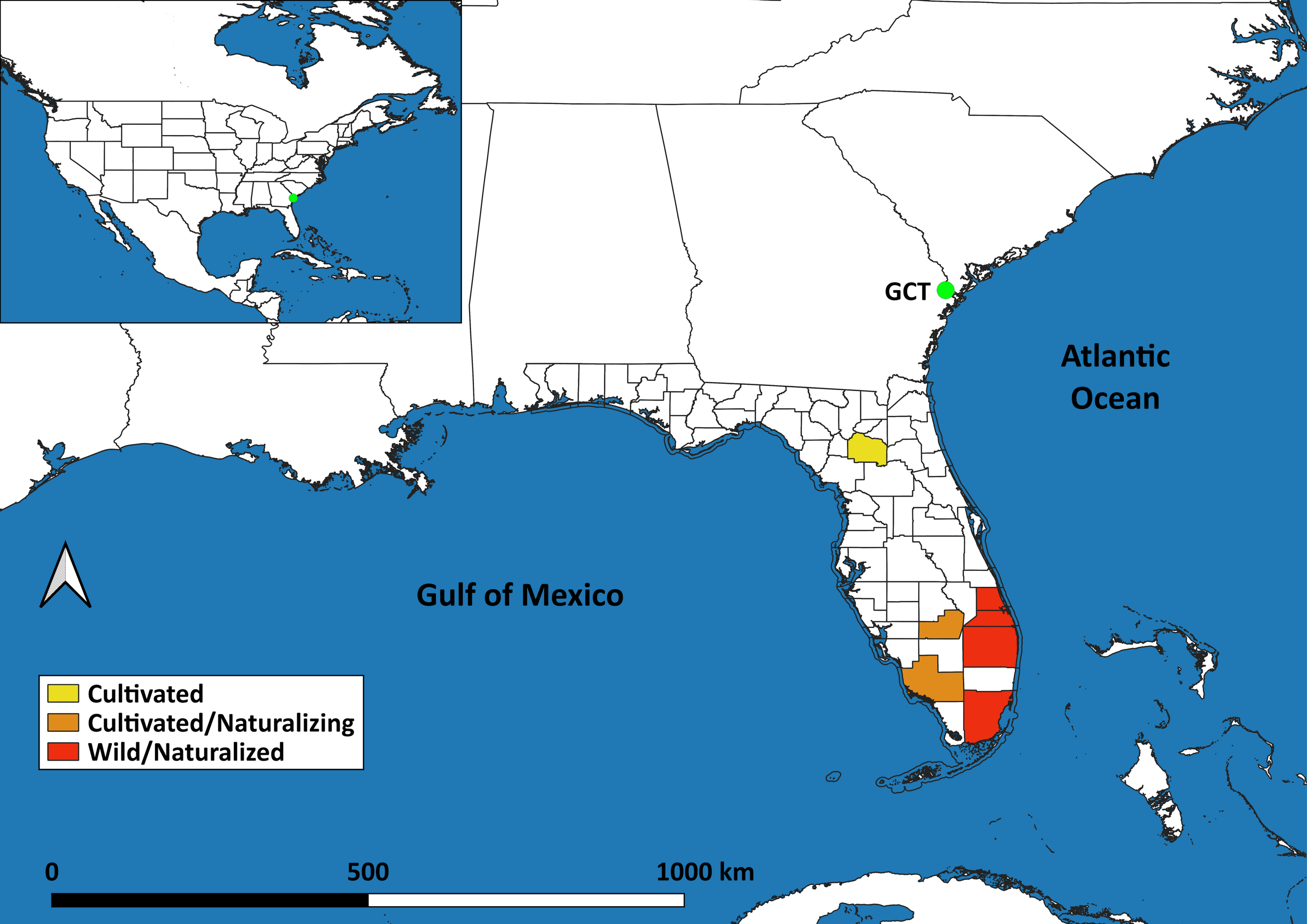


**Supplementary Figure 4.** The distribution of the Federal Noxious Weed (FNW), *Saccharum spontaneum*, distribution in the USA based on imaged herbarium specimen data found in the Symbiota platform through the SERNEC Portal (www.sernecportal.org). Note that this is an update with an expanded distribution from the most recent previous publication^29^. Map generation in the Supplementary Methods and contributing data in Supplementary Table 5.
